# Management and prognosis of patients with brain metastasis from gestational trophoblastic neoplasia: a 24-year experience in Peking union medical college hospital

**DOI:** 10.1186/s12885-015-1325-7

**Published:** 2015-04-28

**Authors:** Changji Xiao, Junjun Yang, Jing Zhao, Tong Ren, Fengzhi Feng, Xirun Wan, Yang Xiang

**Affiliations:** Departments of Obstetrics and Gynecology, Peking Union Medical College Hospital, Chinese Academy of Medical Sciences and Peking Union Medical College, NO.1 Shuaifuyuan Wangfujing Dongcheng District, 100730 Beijing, P. R. China

**Keywords:** Gestational trophoblastic neoplasia, Brain metastasis, FAEV regimen, Prognosis, Risk factor

## Abstract

**Background:**

The optimal treatment for patients with brain metastasis from gestational trophoblastic neoplasia (GTN) has not been established. This study aims to investigate the clinical characteristics and the management of brain metastasis from GTN in relation to patients’ outcomes.

**Methods:**

We retrospectively investigated 109 GTN patients with brain metastasis treated at Peking Union Medical College Hospital from January 1990 to December 2013. Patients mainly received multiagent chemotherapy with florouracil or floxuridine, dactinomycin, etoposide, and vincristine (FAEV) combined with intrathecal methotrexate with or without surgery.

**Results:**

In the 109 patients, sixty-two (56.1%) patients presented for primary therapy and 47 patients had failed chemotherapy elsewhere. Eight early demise patients who died before or during first cycle of chemotherapy were excluded from analysis. The median follow-up time was 47 months (range 9–180 months). The overall 5-year survival rate (OS) was 71.1%, while the OS rate for patients receiving primary chemotherapy in our hospital was 85.5%, and this fell to 51.9% in patients with failure multidrug chemotherapy elsewhere. Multivariate analysis demonstrated that International Federation of Gynecology and Obstetrics (FIGO) scores over 12 (Hazard ratio-HR 1.279, 95% CI 1.061-1.541, *P* = 0.010), failure of previous multidrug chemotherapy (HR 3.177, 95% CI 1.277-7.908, *P* = 0.013), and concurrent renal metastasis (HR 2.654, 95% CI 1.125-6.261, *P* = 0.026) were the risk factors of overall survival in patients with brain metastases from GTN.

**Conclusions:**

Patients with brain metastasis from GTN have favorable outcome by multidrug chemotherapy and adjuvant therapies. Nevertheless, the prognosis is poor if the patients had previous multidrug failure chemotherapy history, concomitant with renal metastasis, or FIGO score over 12. Initial treatment with FAEV combined with intrathecal methotrexate chemotherapy can bring bright prospect to patients with brain metastases from GTN.

## Background

Gestational trophoblastic neoplasia (GTN) is used to refer to a group of uncommon malignant gynecological tumors arising from trophoblastic cells, including invasive mole, choriocarcinoma, placental site trophoblastic tumor, and epithelioid trophoblastic tumor [1]. Owing to their remarkable sensitivity to chemotherapy, the cure rates are almost 100% in the low-risk group and nearly 90% in the high-risk group with current chemotherapy regimens [2,3]. But the prognosis of certain patients with GTN is still poor, including those with far-advanced disease at presentation, and long interval time from the antecedent pregnancy [4]. In addition, brain metastasis was also regarded as a poor prognostic factor in previous reports [5].

Although brain metastasis from GTN is a rare event with an incidence of 3% to 21.4% and about only 222 cases documented in the literature [5,6], the survival rates of patients with brain metastasis are significantly reduced as low as 35-60% [5,7,8]. However, with the rarity of brain metastasis from GTN, there are still no guidelines on treatment strategies for these patients yet. Currently available data are derived from some retrospective reviews including few patients [5,7-10]. While the main treatment strategies of these reports were systemic chemotherapy combined with whole-brain radiation therapy. Given the intellectual impairment by whole-brain radiation therapy over long term in patients, the study of new effective strategies with limited toxic effects have become an intense focus of clinical physicians.

In China, fuorouracil (5-FU) or floxuridine (FUDR)-based combination chemotherapy (florouracil/floxuridine, dactinomycin, etoposide, and vincristine, FAEV) was favorable in the management of high-risk GTN in the Peking Union Medical College Hospital (PUMCH) for several decades [11-13]. Furthermore, our earlier report demonstrated that FAEV regimen was also an effective regimen with manageable toxicity for patients with relapsed/chemoresistant GTN [14]. Therefore, FAEV combined with intrathecal methotrexate chemotherapy is the preferred treatment strategy for the patients with brain metastasis from GTN in PUMCH. In the present study, we collected clinical datas of 109 GTN patients with brain metastasis in our hospital from January 1990 to December 2013, and retrospectively analyzed the management, the prognosis and related risk factors of patients with brain metastasis in GTN.

## Methods

### Data collection

From January 1990 to December 2013, there were 3,209 patients with GTN treated at PUMCH. Patients with brain metastases were identified and reviewed according to the International Federation of Gynecology and Obstetrics (FIGO) criteria for GTN [15]. The diagnostic procedures were computed tomography (CT) or preferably magnetic resonance imaging (MRI) scan of the brain, determination of baseline serum human chorionic gonadotropin(hCG) level, especially the serum β-hCG level, and when applicable, cerebrospinal fluid (CSF) hCG: serum hCG ratio. Approval for this study was obtained from the PUMCH Research Ethics Committee. And written informed consents was obtained from the patients for publication of this retrospective review.

### Treatment protocol

On admission, all patients who were highly suspected with brain metastasis have an initial assessment with detailed history, physical examination, routine blood test, biochemistry and serum hCG level test, X-ray or CT scan of chest and B type ultrasound or MRI scan of pelvis, and CT or MRI scan of brain. And during the treatment, β-hCG level, routine blood test, and serum biochemical examination, were monitored weekly for response and toxicity.

Patients received a combination of systemic chemotherapy and intrathecal injection of methotrexate. According to the previous protocol of chemotherapy, several different chemotherapy regimens were used. FAEV was used in the patients who didn’t receive the 5-FU or FUDR-based combination chemotherapy before[13,14]. If drug resistance had developed or the reduction of the serum β-hCG level was unsatisfactory [14], a replacement regimen of alternating etoposide, methotrexate, dactinomycin (EMA) with cyclophosphamide and vincristine (CO), or alternating EMA with etoposide and cisplatin (EP) was used. If patients remained refractory, almost all salvage regimens were platinum-based. Once the serum β-hCG level was normal, intrathecal injections were stopped, but patients still received an additional 2 to 4 courses of consolidation systemic chemotherapy. The majority of patients required granulocyte colony-stimulating factor because of blood toxicity of chemotherapy.

For the patients with chemotherapy-resistant GTN or with high intracranial pressure which was secondary to hemorrhage, edema, and tumor volume, adjuvant surgical procedures including hysterectomy, pulmonary resection and craniotomy, were used to remove foci of chemotherapy resistant disease or reduce intracranial pressure.

### Assessment of curative effect

Complete remission (CR) was defined as normal β-hCG levels in at least four consecutive weekly determinations. A partial remission (PR) was defined as serum β-hCG levels decreased more than 50% or tumor diminished by more than 50% compared with the pretreatment. Progression of the disease (PD) was defined as the serum β-hCG levels continuing plateau/elevated, or appearance of new metastases for at least two consecutive cycles of chemotherapy [14].

### Statistical analysis

Statistical analyses were performed with SPSS 17.0 statistical software (SPSS, Inc., Chicago, IL). Survival was measured from the date of diagnosis to the date of last follow-up or death. Cases alive and lost to follow-up at the end of the follow-up period were considered censored observations. The overall survival was plotted according Kaplan-Meier method, and the univariate log rank test was used to evaluate the significance of prognostic factors for survival. Multivariate analysis using Cox proportional regression method was performed for the covariates selected in univariate analysis. *P* <0.05 was considered to be statistically significant.

## Results

### Patient characteristics

A total of 109 GTN patients were identified as having brain metastasis at PUMCH in the past 24 years, representing 3.4% of all patients treated with this cancer at our hospital during the same period. Among the 109 patients, 62 patients (56.1%) received primary treatment in our hospital and the rest 47 patients (43.9%) had failed multidrug chemotherapy elsewhere.

Table 1 showed detailed patients characteristics. The median age of the patients was 28 years (range 20–56 years). The most common antecedent pregnancy was mole in 41 (40.6%), followed by term delivery in 36 (35.6%) and non-molar abortion in 24 (23.8%). 69 patients (63.3%) had interval time more than one year. The median pretreatment serum β-hCG levels were 19224mIU/mL (range 62-3049000mIU/mL) before receiving treatment in our hospital. According to FIGO staging and scoring system for GTN in 2000 [16], the most of patients belonged to high-risk group (95.4%), with a median FIGO score of 13 (range 5–23 points).

**Table 1:** **Clinical characteristics of all GTN Patients with Brain Metastasis**

Characteristic	Patient(n = 109)
Age (years), median (range)	28(20–56)
Gravidity, median (range)	2(1–8)
Parity, median (range)	1(0–4)
Interval time(mos.) from antecedent pregnancy median (range)	20(1–288)
Antecedent pregnancy	
Abortion	24(23.8%)
Mole	41(40.6%)
Term	36(35.6%)
No. of metastases	
1 ~ 4	86(78.9%)
5 ~ 8	17(15.6%)
>8	6(5.5%)
Site of metastasis^a^	
Brain metastasis	75(68.8%)
Brain + liver metastasis	12(11.0%)
Brain + kidney metastasis	10(9.2%)
Brain + liver + kidney metastasis	4(3.7%)
Other sites^b^	8(7.3%)
Multidrug chemotherapy history	47(43.9%)
FIGO, median (range)	13(5 ~ 23%)
Pretreatment serum β-hCG level (mIU/mL), median (range)	19224(62–3049000)
FAEV regimens	
Primary treatment	62(56.1%)
Salvage treatment	47(43.9%)

FIGO: International Federation of Gynecology and Obstetrics; hCG: human chorionicgonadotropin.

FAEV: florouracil/floxuridine, dactinomycin, etoposide, and vincristine,

^a^ Lung metastases excluded;

^b^ Including spinal cord, bladder, adrenal gland, intestinal tract, skin and bone.

The distant metastatic sites that were thought to be artery metastasis included the brain, liver, kidney, and several unusual sites such as the bladder, spinal cord, intestinal tract, adrenal gland, skin and bone. Besides lung metastasis, 75 patients (68.8%) presented with isolated brain metastases, 25 patients (22.9%) displayed double-site distant metastases, 6 (5.5%) patients presented triple-site distant metastases, and 3 patients (2.8%) displayed more than 3 sites of distant metastases.

Eight early demise patients who died before or during first cycle of chemotherapy were excluded from the survival and prognostic factors analysis. All of these patients had large-volume disease reflected by very high FIGO scores and multiple metastases (Table 2). What’s more, apart from one patient with recurrent choriocarcinoma, almost all of the early-death patients were delayed in the local hospitals before the definitive diagnosis was made.

**Table 2:** **The clinical characteristics in 8 patients who died before or during first cycle of chemotherapy**

Case No.	Age(yrs)	FIGO score	AP	Interval time (mons)	Pretreatment serum β-hCG level	Site of metastases	Chemotherapy	Cause of death
1	53	15	Abortion	180	99000.0	Lung/brain/kidney	Not done	Brain hemorrhage, respiratory failure
2	30	21	Abortion	10	906720.0	Lung/brain/liver/kidney	Not done	Septic shock
3	31	8	Mole	32	80.0	Lung/brain	5-FU*1d	Brain herniation
4	25	12	Mole	27	57380.0	Lung/brain	EMA*1	Brain herniation
5	32	12	Term	6	200000.0	Lung/brain/kidney	FAEV*2d	Respiratory failure, cardiac arrest
6	36	13	Mole	56	9306.0	Lung/brain/bladder	FAEV*1d	Multiple organ failure
7	26	15	Term	3	60436.5	Lung/brain	FAEV*3d	Brain stem hemorrhage
8^a^	33	17	Mole	36	2400.0	Lung/brain	FAEV*4d	Brain herniation

AP: antecedent pregnancy; EMA: etoposide, methotrexate, dactinomycin; FAEV: floxuridine, dactinomycin, etoposide, and vincristine; 5-FU: fluorouracil.

^a^ recurrent choriocarcinoma.

### Treatment

All patients received multiagent chemotherapy with FAEV combined with intrathecal methotrexate chemotherapy at least two cycles. For the FAEV regimens as the primary treatment group, 16 patients (28.0%) discontinued FAEV therapy because of no response(13 cases) or toxic effects(3 cases). While the salvage treatment group, 24 patients (54.5%) discontinued FAEV therapy because of drug resistance(17 cases) or toxic effects(7 cases). Of 40 patients with no response or toxic effects, 18 patients achieved CR by further salvage chemotherapy with or without surgeries.

Of the 109 patients, 68 patients (62.4%) received 85 times of surgical treatment. Among these patients, 15 (13.8%) underwent pelvic operation (hysterectomy or uterine lesions resection), 18 (16.5%) underwent lung surgery (lobotomy or lung lesion resection), 16 (14.7%) underwent craniotomy, 9 (8.3%) underwent pelvic operation plus lung surgery, 6 (5.5%) underwent lung surgery plus craniotomy, and 2 (1.8%) underwent pelvic operation plus craniotomy. Other surgeries included resection of unilateral adrenal metastasis (1 case) and partial resection of the intestine (1 case). Only two patients received brain irradiation.

### Outcome and survival

Excluding the 8 early-deaths patients, 71 (70.3%) of the remaining 101 patients achieved CR after the comprehensive treatments, 10 (9.9%) patients obtained PR, and 20 (19.8%) patients exhibited PD. Among these 101 patients there were 26 (25.7%) patients died after initial treatments. Most patients (22/26) died of intracranial hemorrhage or with concurrent herniation and multiple organ failure. In the other 4 patients, 3 died of septic shock resulting from myelosuppression during treatments, and another one died of respiratory failure. Of the 57 patients who received primary treatment in our hospital, 48 (84.2%) patients achieved CR; among the 44 patients who were treated secondarily in our hospital, only 23 (52.3%) patients achieved CR.

**Figure 1:**
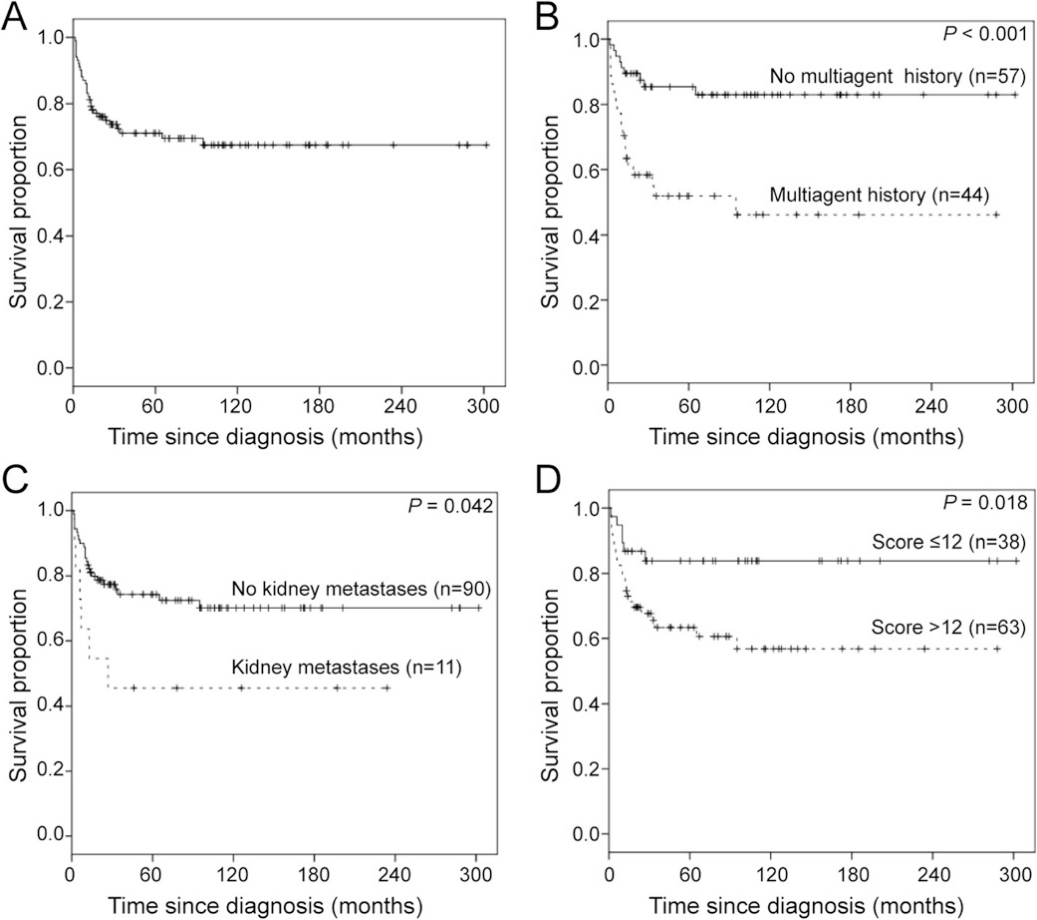
Kaplan-Meier curve for **(A)** overall survival of patients of GTN with brain metastases (n = 101) excluding 8 early-death patients, **(B)** survival of patients without versus with previous multidrug chemotherapy failure history, **(C)** survival of patients with isolated brain metastases versus concurrence of kidney metastases, **(D)** survival of patients with FIGO score ≤12 versus > 12.

Follow-ups were provided to all survival patients. The median follow-up time was 47 months (range 8–180 months), but 6 patients lost follow-ups. Among these patients, 9 (11.3%) patients relapsed within 3 to 84 months after completion of the initial treatment, most of whom (6/9) relapsed in the first year, and 4 of them exhibited distant metastasis and died of PD during the secondary treatments. 9 (12.7%) patients obtained 11 times pregnancy and achieved 10 live births. Therefore, a total of 30 patients died during the initial treatments or after recurrence. The overall five-year survival (OS) rate of all patients was 71.1% (Figure 1A). However, the OS for patients who received their primary treatment in our hospital was 85.5%, and this fell to 51.9% in patients transferred to us from other centres with multidrug chemotherapy failure history (*P* < 0.001).

### Prognostic variables

Univariate analysis showed that age (*P* = 0.020), interval time from antecedent pregnancy (*P* = 0.018), number of distant metastatic sites (*P* = 0.030), multi-agent chemotherapy failure history (*P* < 0.001, Figure 1B), concurrence with liver (*P* = 0.013) or kidney metastases (*P* = 0.042, Figure 1C), craniotomy (*P* = 0.029), and FIGO score (*P* = 0.018, Figure 1D), were associated with prognostic significance (Table 3).

**Table 3:** **Univariate analysis of prognostic factors in patients with brain metastasis of GTN**

Clinical Factors	Survival rate (%)	*P*value
Age		
<40(n = 92)	73.9	
≥40(n = 9)	33.3	0.020
Antecedent pregnancy		
Abortion(n = 24)	50	
Mole(n = 36)	75	
Term(n = 41)	78	0.063
Interval time(mos.) from antecedent pregnancy		
<13(n = 38)	84.2	
≥13(n = 63)	61.9	0.018
No. of metastasis		
≤4(n = 86)	70.9	
>4(n = 15)	66.7	0.461
Pretreatment serum β-hCG level		
<100,000(n = 74)	68.9	
≥100,000(n = 27)	74.1	0.576
No. of distant metastatic sites^a^		
1(n = 71)	78.9	
≥2(n = 30)	50	0.030
No. of multi-agent chemotherapy failure history		
No(n = 57)	84.2	
Yes(n = 44)	52.3	<0.001
Liver metastasis		
No(n = 86)	74.4	
Yes(n = 15)	46.7	0.013
Kidney metastasis		
No(n = 90)	73.3	
Yes(n = 11)	45.5	0.042
Pelvic surgery^b^		
No(n = 75)	74.7	
Yes(n = 26)	57.7	0.082
Lung surgery^c^		
No(n = 68)	67.6	
Yes(n = 33)	75.8	0.359
Craniotomy		
No(n = 77)	66.2	
Yes(n = 24)	83.3	0.029
FIGO score		
≤12(n = 38)	84.2	
>12(n = 63)	61.9	0.018

^a^ Including brain, liver, kidney, and several unusual sites, e.g. the spinal cord, bladder, adrenal gland, intestinal tract, skin and bone;

^b^ hysterectomy or uterine lesions resection;

^c^ lobectomy or lung lesion resection.

Multivariate analysis demonstrated that FIGO score over 12 (Hazard ratio-HR 1.279, 95% CI 1.061-1.541, *P* = 0.010), failure of previous multidrug chemotherapy (HR 3.177, 95% CI 1.277-7.908, *P* = 0.013), and concurret with renal metastasis (HR 2.654, 95% CI 1.125-6.261, *P* = 0.026) were independently significant for poor survival (Table 4). While, the presence of the liver metastases was not an independent poor prognostic factor (HR 1.681, 95% CI 0.749-3.149, *P* = 0.208).

**Table 4:** **Multivariate analysis of the prognosis of the patients with brain metastasis from GTN**

Variable	Hazard ratio	95% CI	*P*value
Age	0.987	0.938-1.039	0.611
Interval time (mos.) from AP	0.564	0.147-2.158	0.403
Multi-agent failure therapy	3.177	1.277-7.908	0.013
Liver metastasis	1.897	0.787-4.573	0.154
Kidney metastasis	2.654	1.125-6.261	0.026
Craniotomy	1.835	0.565-5.962	0.312
FIGO score	1.279	1.061-1.541	0.010

## Discussion

As the first curable solid tumor by chemotherapy, the cure rate of patients with GTN is more than 90% by the current chemotherapy regimens [17]. However, there are still a small proportion of the patients who die from treatment failure, especially for those with distant metastasis. Brain is the most common distant artery metastatic site of GTN. In the present study, the patients with brain metastasis presented a death rate of 29.7%, which was much higher than the reported 5% overall death rate of GTN [18]. Therefore, effective management of the patients with brain metastasis from GTN remains a clinical challenge.

Due to the rarity of the GTN with about 222 cases documented in the literature and the much less occurrence of the brain metastasis from GTN, most published data have been obtained from studies with small number patients [5,7,8,10,19-25]. In this retrospective analysis, we collected 109 cases of brain metastasis in GTN during a 24-year period. To the best of our knowledge, this study is the largest number of patients to explore the management, clinical outcomes and relevant risk factors associated with prognosis of GTN patients with brain metastasis.

In the latest review about brain metastases from GTN [6], the outcome of patients with brain metastases from GTN was improved with multimodal therapy including craniotomy, whole brain radiotherapy, and EMA-EP or EMA-CO chemotherapy. Nonetheless, brain metastasis from GTN is a grave disease with a median survival time of about 12 months from diagnosis of brain metastasis. Our results showed that GTN metastastic to brain was curable if the patients did not have an early death, with the overall five-year survival rate of 71.1%. The aggressive chemotherapy is the first choice for these patients. In view of our experience with chemotherapy for GTN, 5-FU (before 2000 years) or FUDR (since 2000 years)-based combination chemotherapy was favorable in the management of GTN in our hospital for half of a century [13,14,26]. In our center, 5-FU, dactinomycin, vincristine (FAV) is the preferred combined chemotherapy for patients with high-risk GTN. According to the accepted guidelines that have a different mechanism of action in selecting agents for combination chemotherapy, etoposide is a useful anticancer agent and has a different mechanism of action to 5-FU and dactinomycin. Moreover, etoposide is included in available chemotherapy regimens for high-risk GTN in vast literatures [1,27-29]. Therefore, FAEV combined with intrathecal methotrexate chemotherapy is the first-line chemotherapy for the patients with brain metastases from GTN in PUMCH. Furthermore, our earlier report the FAEV is also an effective regimen with manageable toxicity for patients with relapsed/chemoresistant GTN[14]. Thus, it is also used as second-line or third-line treatment for the patients treated secondarily in our hospital. In this study, for the 57 patients in the FAEV regimens as the primary treatment group, 41 patients (71.9%) achieved CR. This strongly suggested that primary treatment with FAEV combined with intrathecal methotrexate can produce good outcomes in brain metastatistic GTN. However, becase of salvage therapy being more likely to fail in heavily pre-treated patients [14], in the salvage treatment group, only 20 patients (45.5%) achieved CR. During the follow-up time, there were only 9 patients (12.6%) relapsed and 4 of them were still alive at the time of analysis. 9 patients (12.7%) obtained 11 times pregnancy and achieved 10 live births. Hence, these data clearly confirmed that the patients with brain metastasis GTN could have a good outcome with systematic chemotherapy plus intrathecal injection of methotrexate.

Time is of the essence in the management of GTN, and it’s crucial to realize that the patients should receive early life-saving and standard therapy, preferably in specialist centers. In the present study, eight patients (7.3%) with large tumor burden of disease died before or during first cycle of chemotherapy. This is because of considerable delay in the diagnosis and the substandard treatment in the local hospital. Therefore, delay in the initiation of treatment could adversely affect the prognosis, and early life-saving treatment such as craniotomy is quite necessary for these patients. Among the 101 patients, there were 24 patients (22.0%) received craniotomy, 15 of whom were emergency craniotomy because of the life-threatening high intracranial pressure secondary to hemorrhage and edema. After treatment, 14 patients (93.3%) achieved CR in the 15 cases. But only 6 patients (66.7%) achieved CR for the remaining 9 patients who received craniotomy either because of the brain metastases as the primary clinical manifestations of GTN or to remove the foci of chemotherapy resistant disease. Indeed, the univariate analysis showed the prognosis in patients who received craniotomy were significantly improved (*P* = 0.029). This strongly suggested that the craniotomy play a role in selected patients with brain metastases from GTN, especially in patients who display rapidly deteriorating signs, because this operation can save the patient’s life and help to win time for them to receive subsequent systemic chemotherapy. In addition, a failed prior chemotherapy history was the single important poor prognostic factor for survival, especially for those who received nonstandard treatment before transferred to our hospital. The substandard chemotherapy not only delayed the duration of the disease which would lead to the widespread of the disease, but also easily developed to chemoresistant and recurrent disease. The multivariate analysis demonstrated that previous multidrug chemotherapy failure history (HR 3.177, 95% CI 1.277-7.908, *P* = 0.013) was an independent poor prognostic indicator for survival. Actually, the OS of the patients treated secondarily in our hospital was 51.9%, much less than 85.5% of patients who received the primary treatment in our hospital (univariate analysis, *P* < 0.001). This result is also supported by the previous studies [5,13,30]. Therefore, in order to improve the survival of patient with brain metastasis of GTN, early diagnosis and standard treatments should be promptly applied at specialist centers to prevent the development of drug resistance and multiorgan metastasis.

Apart form the two patients who had stereotactic radiotherapy, we seldom use the cranial radiation therapy since it can induce long-term intellectual impairment in patients who are cured [10]. Since the effect on tumor vasculature from radiotherapy is a late phenomenon, we do not think that the use of routine cranial radiation to reduce the chance of brain hemorrhage is appropriate. In fact, the cerebral hemorrhage of these patients is often an early stage event. Some of them even firstly present with intracranial hemorrhage and high intracranial pressure. In this time, emergency craniotomy plays a key role in saving the patient’s life. In fact, our univariate analysis results showed that craniotomy was an favorable prognostic factor (*P* = 0.029). And in the 15 cases with emergency craniotomy, 14 patients (93.3%) achieved a CR. These results demostrate that it is possible to have a good outcome with chemotherapy and craniotomy in selected cases.

It is recognized that the interval time between antecedent pregnancy and treatment, the presence of liver metastasis, and treatment with substandard chemotherapy were related to the prognosis of GTN patients with brain metastasis [5,30]. However, four new features were found relevant to poor outcome: more than one distant metastatic site, concurrence with kidney metastasis, FIGO score over 12, and age more than 40 years (Table 3). Although the number of distant metastatic sites was only found to be significant in the univariate analysis (*P* = 0.030), this result may still be important because of widespread nature of the disease. Differently from the reported poor prognosis of patients with coincidence of liver metastasis in GTN [29-31], our multivariate analysis showed that concurrence with the liver metastases was not an independent significant predictor of poor outcome. Instead, the presence of renal metastasis (*P* = 0.026) was an independently significant prognostic indicator (Table 4). Indeed, the five-year survival in patients with brain metastasis and kidney metastasis was 45.5%, much lower than 74.3% in the patients with brain metastasis only (Figure 1C). FIGO score over 12 (*P* = 0.010) is another independent poor prognostic indicator, which is quite similar with other reported metastatic site of GTN [13,32].

Our previous study suggested that FAEV regimen was an effective regimen with manageable toxicity for high-risk GTN patients; and the major restrictive adverse event of FAEV regimen was hematologic toxicity [12,14]. In the present study, the regimen was tolerated well and the toxic effects were similar to that observed in our previous study [12,14] (data not shown). Other common adverse events to FAEV regimen were nausea and vomiting, which were easily circumvented with the use of standard antiemetics and sometimes in combination with corticosteroids.

## Conclusions

In conclusion, we have shown that the patients with brain metastases from GTN were curable with the use of combined systemic combination agent chemotherapy plus intrathecal methotrexate with or without additional surgeries in selected cases. FAEV regimens combined with intrathecal MTX chemotherapy can produce favorable outcomes for GTN patients with brain metastasis who were treated primarily. The prognosis for patients with age over 40 years, presence of kidney metastasis, previous multidrug chemotherapy failure history, multiple-site distant metastasis, and FIGO scores over 12 was poor. Further work needs to be done to improve the prognosis. For women of childbearing age presenting with unexplainable lung or brain metastases, a diagnosis of GTN should be considered and serum hCG measured as part of the initial work-up. The best outcomes are achieved when such patients are transferred to major GTN centers for specialised management delivered by experts.

## Abbreviations

GTN: Gestational trophoblastic neoplasia
FAEV: Florouracil/floxuridine, dactinomycin, etoposide, and vincristine
PUMCH: Peking Union Medical College Hospital
FIGO: International Federation of Gynecology and Obstetrics
CT: Computed tomography
MRI: Magnetic resonance imaging
hCG: Human chorionic gonadotropin
CSF: Cerebrospinal fluid
EMA: Etoposide, methotrexate, and dactinomycin
CO: Cyclophosphamide and vincristine
EP: etoposide and cisplatin
CR: Complete remission
PR: Partial remission
PD: Progression of the disease
